# Epithelioid Hemangioendothelioma of Tongue: A Rare Presentation

**DOI:** 10.30476/dentjods.2023.96208.1927

**Published:** 2023-09

**Authors:** Durga Bai Yendluri, Chandrika Chinta, Chiranjeevi Vedula

**Affiliations:** 1 Dept. of Periodontics, Government Dental College and Hospital, Afzalgunj, Hyderabad, India; 2 Former Post Graduate, Dept. of Periodontics, Government Dental College and Hospital, Afzalgunj, Hyderabad, India; 3 Tirumala Institute of Dental Sciences and Research Centre, Nizamabad, India

**Keywords:** Hemangioendothelioma, Epithelioid hemangioendothelium, Immunohistochemistry, Tongue

## Abstract

Hemangioendothelioma is a diverse set of proliferative and neoplastic vascular lesions with biological characteristics that fall halfway between benign hemangioma and malignant angiosarcoma. Hemangioendothelioma of the oral cavity is extremely rare and if present, it is seen on lips, gingiva, tongue, maxilla, and mandible. The following case report is about a lesion on the right ventrolateral border of the tongue of a six-year-old female patient. A Laser excision was done. Histopathology revealed the features of hemangioendothelioma. An immunohistochemical (IHC) study was done to correlate the findings with a histopathological picture and arrived at the final diagnosis of epithelioid hemangioendothelioma (EHE). The patient was followed up for two years and no recurrence was noticed.

## Introduction

Weiss and Enzinger [ 1 ] were the first to define epithelioid hemangioendothelioma (EHE), a rare borderline vascular tumour characterized by endothelial cells with an epithelioid shape. These tumors have the potential for local recurrence and metastasis at a lower rate than conventional angiosarcomas [ 2 ]. According to the WHO, EHE is an extremely rare, low-grade malignant vascular tumour with possible metastases that accounts for 1% of all vascular tumors [ 3 ]. In the oral cavity, only a few occurrences were recorded in the literature. In terms of clinical presentation, it can resemble reactive lesions like pyogenic granulomas, chronic periodontal disease, and peripheral giant cell granulomas. Typically, patients have an ulcerated soft tissue mass that resembles friable granulation tissue initially [ 4 ]. The hard palate is a rare intraoral location, with the submandibular area, gingiva, and alveolar mucosa being the most frequent ones. Histologically, hemangioendothelioma is distinguished by the proliferation of round, eosinophil-infiltrated endothelial cells, vacuolation of the cytoplasm, and myxohyaline stroma. Based on histological criteria, it is characterized as kaposiform, Dabskos, or epithelioid. Histologically, EHE is distinguished by the proliferation of round, eosinophil-infiltrated endothelial cells, cytoplasm vacuolation, frequent angiocentric inflammation, and myxohyaline stroma [ 5 ]. We present a case of EHE that manifested as a painless enlargement on the tongue and was provisionally diagnosed as a fibroma. Histopathological and immunohistochemical (IHC) findings supported the diagnosis of EHE. 

## Case Presentation

**Figure 1:**
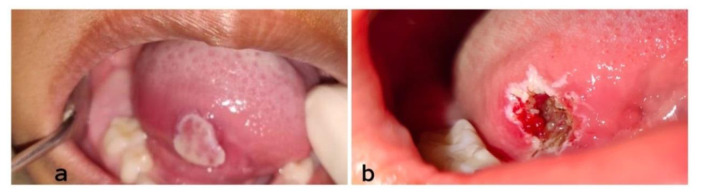
**a:** Lesion on the right ventrolateral surface of the tongue, **b:** Immediately after excision

**Figure 2:**
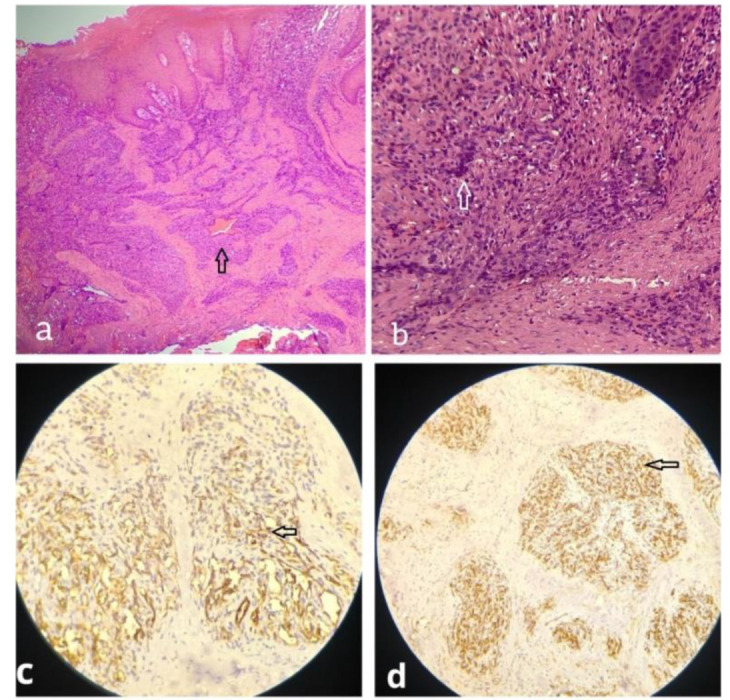
Histological examination showed nests of cells surrounding irregular vascular channels (**a:** H&E stain) with spindling and hyperchromatism (**b**: H&E stain), **c:** Immunohistochemical staining- Positive tumor cells cytoplasmic reaction for d: CD 31 and CD 34

A six-year-old female patient presented to the Department of Periodontics with a chief complaint of growth on the right lateral surface of the tongue which was lasting for more than 10 days. The patient gave a history that the growth was smaller initially, gradually increased and attained present size. The patient had no significant medical history. On intraoral examination, a solitary growth was noticed on the right ventrolateral surface of the tongue, which was 3 cm away from the tip having an irregular shape (Figure 1). The lesion was sessile with a yellowish-white surface, ulcerated along the borders, measuring about 1.5cm by 0.7cm, non-indurated and firm in consistency. No bleeding or tenderness was seen associated with the lesion. A provisional diagnosis of fibroma was made based on these clinical findings. The lesion was surgically excised under local anaesthesia with the help of a diode laser and sent for microscopical evaluation. Histopathological examination of hematoxylin and eosin stained sections (Figure 2a and b) showed a fibrocellular connective tissue stroma with solid nests and sheets of polygonal cells separated by hyalinized stroma. These solid nests of cells were polygonal to polyhedral with hyperchromatic nuclei, scanty cytoplasm resembling epithelioid cells and seen surrounding irregular vascular channels, with the lining endothelial cells showing pleomorphism and hyperchromatism. In some places, these nests of cells were pleomorphic and showed spindling. The overlying epithelium was parakeratinized stratified type. These microscopic features revealed the lesion as EHE. An IHC study was done to confirm the diagnosis. IHC stains for CD31 and CD34 exhibited cytoplasmic positivity of epithelioid tumour cells, confirming the lesion as EHE (Figure 2c and d).

The patient was referred to a general physician for needful systemic examination and confirmed that no abnormalities were noticed systemically as well. She was followed up for two years, and no recurrence was found. 

## Discussion

**Figure 3:**
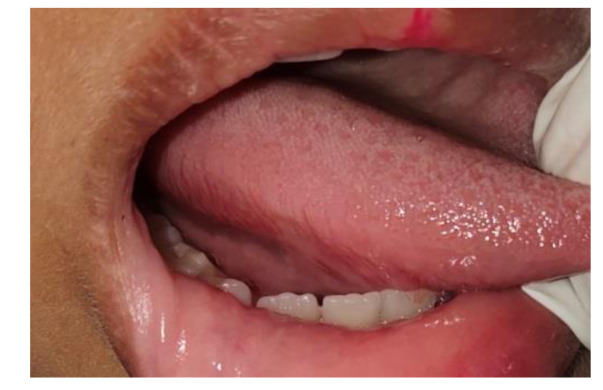
Follow-up at 2 years

Mallory [ 6 ] coined the word hemangioendothelioma in 1908 to describe all the proliferations arising from endothelial cells. Endothelial cell growth surrounding a vascular lumen characterizes hemangioendothelioma [ 7 ]. It is classified as a vascular neoplasm having a low-to-intermediate malignant potential. Its clinical and pathological characteristics place it somewhere between hemangioma and traditional angiosarcoma. EHE of the tongue was infrequently reported. There have only been ten occurrences of it on the tongue that have been documented in the literature [ 8 ]. The clinical appearance of it was nonspecific, most often appearing as a benign, painless lump, while the lesion could occasionally be ulcerated [ 9 ]. The present case was found to be similar in that it had ulcerated borders. The cause of EHE is unknown; however, Tanas RM *et al*. [ 10 ] postulated that the fusion of two distinct genes, WWTR1, and CAMTA1, is caused by the reciprocal translocation of chromosomes 1 and 3. Microscopically, it is distinguished by the proliferation of vascular epithelioid type endothelial cells with intracytoplasmic vacuoles; a few of the vacuoles may contain erythrocytes and invasion of the epithelial cells in underlying muscle tissue may or may not be present [ 11 ]. Most of the time, it is discovered that the connective tissue stroma is fibro-vascular and has many vascular channels. Spindle-shaped cells may also be present in large numbers [ 2 ], which was found to be similar in the present case. According to Chi *et al*. [ 12 ], endothelial cells of EHE show positive expression for endothelial markers such as CD34, CD31, and factor VIII in immunohistochemistry. However, in our case, the tumour cells showed positive expression for CD31 and CD34. Histopathological and IHC results are correlated to diagnose EHE. The most effective treatment for oral EHE is broad surgical excision. Laser-assisted excision was done in the present case to minimize bleeding and enhance the comfort of the patient. Nine lesions out of 30 documented cases indicated recurrence, according to a thorough analysis of the literature. The patient was recalled for regular follow-ups due to the lesion’s recurrence rate and malignant potential. The patient was followed up for two years and no recurrence was noticed (Figure 3). 

The authors certify that they have obtained all appropriate patient consent forms. In the form the patient’s parents have given their consent for the images and other clinical information to be reported in the journal.

## Conclusion

Based on the case and the literature reviewed, hemangioendothelioma of the oral cavity is a rare occurrence that is frequently mistaken as a benign reactive lesion, necessitating a histological diagnosis. Due to a lack of specific diagnostic criteria, the histological image remains disputed, causing diagnostic hurdles at times. In cases of oral hemangioendothelioma, prompt immunohistochemistry confirmation, followed by surgical treatment, should be required to limit the risk of local recurrence and metastasis.

## Acknowledgment

We would like to thank the Department of Oral Pathology, Government Dental College and Hospital, Hyderabad for the Histopathological analysis.

## Conflict of Interest

The authors declare that they have no conflicts of interests.
